# Prebiotic Galacto-Oligosaccharides Impact Stool Frequency and Fecal Microbiota in Self-Reported Constipated Adults: A Randomized Clinical Trial

**DOI:** 10.3390/nu14020309

**Published:** 2022-01-12

**Authors:** Marieke H. Schoemaker, Jeske H. J. Hageman, Dominique ten Haaf, Anita Hartog, Petra A. M. J. Scholtens, Jos Boekhorst, Arjen Nauta, Rolf Bos

**Affiliations:** 1 FrieslandCampina, 3818 LE Amersfoort, The Netherlands; marieke.schoemaker@frieslandcampina.com (M.H.S.); Jeske.hageman@frieslandcampina.com (J.H.J.H.); dominique.tenhaaf@frieslandcampina.com (D.t.H.); arjen.nauta@frieslandcampina.com (A.N.); 2 NIZO Food Research, 6718 ZB Ede, The Netherlands; anita.hartog@nizo.com (A.H.); petra.scholtens@nizo.com (P.A.M.J.S.); 3 Host Microbe Interactomics Group, Wageningen University & Research, 6708 WD Wageningen, The Netherlands; jos.boekhorst@wur.nl

**Keywords:** galacto-oligosaccharides, stool frequency, stool consistency, stool characteristics, constipation, microbiota

## Abstract

Constipation is a major issue for 10–20% of the global population. In a double-blind randomized placebo-controlled clinical trial, we aimed to determine a dose-response effect of galacto-oligosaccharides (GOS) on stool characteristics and fecal microbiota in 132 adults with self-reported constipation according to Rome IV criteria (including less than three bowel movements per week). Subjects (94% females, aged: 18–59 years) received either 11 g or 5.5 g of Biotis^TM^ GOS, or a control product, once daily for three weeks. Validated questionnaires were conducted weekly to study primarily stool frequency and secondary stool consistency. At base- and endline, stool samples were taken to study fecal microbiota. A trend towards an increased stool frequency was observed after the intervention with 11 g of GOS compared to control. While during screening everybody was considered constipated, not all subjects (*n* = 78) had less than three bowel movements per week at baseline. In total, 11 g of GOS increased stool frequency compared to control in subjects with a low stool frequency at baseline (≤3 bowel movements per week) and in self-reported constipated adults 35 years of age or older. A clear dose-response of GOS was seen on fecal *Bifidobacterium*, and 11 g of GOS significantly increased *Anaerostipes hadrus*. In conclusion, GOS seems to be a solution to benefit adults with a low stool frequency and middle-aged adults with self-reported constipation.

## 1. Introduction

Constipation is one of the most common digestive problems in Western countries and can be defined as difficult, unsatisfactory, or infrequent defecation [1]. Constipation is a major issue for 10–20% of the global population [2,3,4]. It is more common in women than in men and more frequent in older adults [5]. Symptoms may be acute (less than a week) or chronic (for at least three months) and have a significant impact on daily living and well-being [6,7]. Due to its high prevalence and difficult management, constipation generates high costs in health care systems every year [6]. The current interventions for constipation include lifestyle and dietary modifications as well as pharmacological interventions with stool softeners, osmotic laxatives, stimulant laxatives, and bulking agents [8,9,10]. Dietary modifications include an increased intake of fiber-rich fruits and vegetables together with a sufficient intake of water. Other factors to be considered are physical activity and stress [10,11].

There is an increasing interest in evidence-based alternatives for the use of laxatives because of undesired side effects of some pharmacological therapies [12]. Some of the studied alternatives are dietary fibers, certain probiotic strains [10], and prebiotic oligosaccharides [12]. Prebiotics are defined as substrates that are selectively utilized by host microorganisms in the gut conferring a health benefit [13]. A few meta-analyses have reported the effects of prebiotics on reducing constipation, indicating that only a low number of trials are available, but these trials show a positive effect [12,14]. Furthermore, prebiotic oligosaccharides have been shown to positively affect stool frequency and stool consistency [12]. The most studied prebiotics are fructo-oligosaccharides, inulin, and galacto-oligosaccharides (GOS) [12,14]. GOS are indigestible carbohydrates that reach the large intestine fairly intact where they serve as substrates for bacterial fermentation to modulate the composition and activity of gut microbiota [15].

There is a growing body of evidence on the health effects of GOS linked to digestive comfort [16,17,18,19,20,21]. A limited number of small-scale studies were performed to assess the effect of GOS on constipation, all in older adults (60–92 years of age). Teuri and Korpela found that a daily intake of 9 g of GOS relieves constipation in elderly people [18]. Surakka et al. performed an intervention in the elderly with yoghurt that included GOS (10 g/day). In that study, it was observed that GOS facilitated defecation and increased the number of Bifidobacteria [21]. In a study by Sairanen et al., it was shown that yoghurt with prunes, linseed, and 12 g of GOS a day also relieved constipation by increasing the defecation frequency [19]. Altogether, these studies give some indication that daily consumption of GOS can relieve constipation, at least in older adults. The effect of GOS on constipation in the general adult population remains unknown. 

Some evidence suggests that dysbiosis of gut microbiota may contribute to constipation, however, it is not clear yet whether specific bacterial species are associated with the onset and/or continuation of constipation [22]. It has been reported that the amount of bifidobacteria is decreased in constipated adults [23,24]. In a study by Parthasarthy et al., the correlation of constipation status with fecal microbiota and mucosal microbiota was studied and constipated patients seemed to have a unique profile of colonic microbiota [25]. Intervention with GOS showed that GOS has a prebiotic effect in healthy adults, obese adults, and adults suffering from IBS, where its effect may rely on increasing bifidobacteria [16,17,26]. In addition, it has been suggested that short-chain fatty acids (SCFA) produced by gut microbiota affect stool characteristics and constipation [22]. As the association between gut microbiota and constipation is still unclear, it would be of interest to investigate the potential impact of GOS.

To further extend the knowledge about the clinical efficacy of prebiotic GOS in the general adult population with constipation, the current study was designed. In a randomized, double-blind, placebo-controlled, parallel study, a daily dose of 11 g of GOS was investigated on stool characteristics, with stool frequency as the primary outcome and stool consistency as the secondary outcome. In addition, the effect of a daily lower dose of 5.5 g of GOS on stool frequency and consistency was examined. As a tertiary outcome of this study, the effect of the interventions on the fecal microbiota composition, and bifidobacteria in particular, was investigated.

## 2. Materials and Methods

### 2.1. Study Design

A randomized, double-blind, placebo-controlled parallel intervention study was conducted. In total, 132 healthy adults with self-reported constipation were included. Inclusion criteria were healthy adults with a BMI between 18.5 and 28.0 kg/m^2^, with self-reported constipation according to the Rome IV criteria with fewer than three bowel movements per week and one or more of the following criteria: straining during at least 25% of all defecations, lumpy or hard stool in at least 25% of defecations, sensation of incomplete evacuation for at least 25% of defecations, sensation of anorectal obstructions/blockage for at least 25% of defecations, or manual maneuvers to facilitate at least 25% of defecation, as indicated in the screening questionnaire. Subjects that had any of the following criteria were excluded from participation: major abdominal surgery interfering with GI function, major comorbidities that may have an impact on the study results, use of medication that may affect the results of the investigational product, use of antibiotics within 90 days before start of the study, use of strict diets, self-reported lactose-intolerance, self-reported cow’s milk protein allergy, diagnosed diabetes, pregnant and lactating women, expected change in lifestyle within 14 days before and during the trial period, history of side effects with the use of prebiotic supplements, use of alcoholic beverages for men >28 units/week and >4/day and for women >21 units/week and >3/day. 

### 2.2. Ethics

This study was approved by the Medical Ethics Review Committee Brabant, the Netherlands (NL73442.028.20), and was registered at the Netherlands Trial Register (NL8758). The study was conducted in accordance with the principles of the Declaration of Helsinki (Fortaleza, Brazil 2013), and the requirements described in the EU Clinical Trials Directive 2001/20/EC transposed in the Revision of the Dutch Medical Research involving Human Subjects Act (WMO, effective as of 1 March 2006).

### 2.3. Study Procedures

Subjects were recruited by advertisements in regional newspapers and on social media like Facebook, Instagram, and Google. Inclusion criteria were clearly mentioned and both male and female subjects were invited. Interested subjects were requested to fill in a short pre-screening questionnaire which was discussed by phone with a trained employee with a medical background. Subjects who passed the pre-screening and were still interested in the study were invited for an information meeting at the research center. During this information meeting, a detailed instruction of the study was provided, and all subjects had the opportunity to ask questions. Subjects willing to participate in the study signed the informed consent form after the information meeting.

During screening, subjects had to fill in a questionnaire, which aimed to assess the eligibility of the subject. This questionnaire included questions about the in- and exclusion criteria and dietary fiber intake.

Subjects were randomly assigned to a treatment group using a random number generator. Stratification according to fiber intake was used. Subjects received a box with sachets containing either 15.1 g of powder with 11 g of Biotis^TM^ GOS (FrieslandCampina, Amersfoort, The Netherlands) or 15.1 g of maltodextrin (control) (Tate&Lyle, Koog aan de Zaan, The Netherlands). To be able to study a dose-response effect, the secondary aim of the study, 7.5 g of powder with 5.5 g of Biotis^TM^ GOS was provided to a third group. To secure blindness of the study team, the closed boxes containing the sachets with powder were corrected in weight. The composition of the products is shown in Table 1. Products were blinded per treatment and three different codes were used. The study team was blinded until database lock was completed.

Subjects were requested to consume the study products in the morning every day for three weeks. The powders had to be dissolved in a drink. Furthermore, subjects were instructed to not change their lifestyle but to maintain their physical activity and habitual food and fluid intake during the study and 14 days before. Subjects were instructed to consume a maximum of two alcoholic beverages per day. All subjects were requested to stop using medications for their constipation and (non-pharmaceutical) drugs during the study.

Subjects were asked to fill in a daily diary to use as input for the weekly questionnaire in which information related to stool frequency and characteristics, bloating and adverse events, changes in daily habits including physical activity, smoking, alcohol, coffee and fluid intake, compliance to the study protocol, and medication intake were recorded. Furthermore, to gain more insight in the stool characteristics and related symptoms, the Constipation Scoring System [27], Bristol Stool Chart (BSS) [28] and PAC-SYM (ACUTE) (Patient Assessment of Constipation Symptoms) (version 4 August 2020, MAPI Research Trust) questionnaires were used. Supplemental Figure S1 illustrates a time diagram of the study procedures and assessments.

### 2.4. Fecal Sampling and Analysis

At the start and the end of the study, fecal samples were collected per subject. Per sample, about 5–8 g of fresh stool was collected. After collection, the samples were frozen in freezers given to the subjects in materials provided for this reason. Samples were brought in during the visits to the study site.

Short-chain fatty acids (SCFA) (lactate, acetate, propionate, butyrate, isobutyrate, valerate, and isovalerate) were extracted and determined by high-performance anion-exchange chromatography with UV and refractive index detection, as previously described by Gommers et al. [29].

DNA isolation, including vigorous bead-beating steps, was performed as described previously [29]. Barcoded amplicons from the V3–V4 region of 16S rRNA genes were generated using a 2-step PCR and according to previously described methods [30]. For the library PCR step in combination with sample-specific barcoded primers, purified PCR products were shipped to BaseClear BV (Leiden, The Netherlands). PCR products were checked on a Bioanalyzer (Agilent, Santa Clara, CA, USA) and quantified. This was followed by multiplexing, clustering, and sequencing on an Illumina MiSeq with the paired-end (2×) 300 bp protocol and indexing. The sequencing run was analyzed with the Illumina CASAVA pipeline (v1.8.3) with de-multiplexing based on sample-specific barcodes. Sequence reads of too low quality (only “passing filter” reads were selected) and reads containing adaptor sequences or PhiX control were discarded from the raw sequencing data. On the remaining reads, a quality assessment was performed using FastQC version 0.10.0 (http://www.bioinformatics.babraham.ac.uk/projects/fastqc/ (accessed on 14 July 2021)).

### 2.5. Statistical Analysis

This study was calculated to have 80% power at a two-sided *p*-value of 0.05 to detect a difference in stool frequency of 1 bowel movement per week, with an estimated standard deviation of 1.5 with 111 subjects included in total. Taking into account an expected drop-out rate of 15%, a total of 132 subjects, 44 per group, were included.

Statistics were performed with SAS statistical package, Studio version 3.7, and were predefined in a statistical analysis plan. To answer the primary question of this study, a mixed model repeated measures analysis using a Poisson distribution with treatment and visit as fixed effects (11 g vs. control at endline), treatment*visit as fixed interaction effect, and baseline BMI, baseline stool frequency, and fiber intake category as covariates was performed. Subgroup analyses on two different age groups (younger adults: 18–35 years of age and middle-aged adults: ≥35 years of age) and subjects with actual constipation at baseline (<3 bowel movements per week) were performed. As the number of subjects with less than 3 bowel movements per week at baseline (*n* = 54) was much lower than expected based on screening information, a post-hoc analysis on subjects with a low stool frequency (≤3 bowel movements per week) (*n* = 98) was performed as well. To answer the secondary questions on the other outcomes and the 5.5 g GOS group, a similar model with treatment and visit as fixed effects, treatment*visit as fixed interaction effect, and baseline BMI, baseline stool frequency, and fiber intake category as covariates was performed.

The outcomes of the Bristol stool chart were compared with a Wilcoxon rank-sum test. The constipation scoring system and PAC-SYM questionnaire outcomes were analyzed and SCFA were analyzed with an ANCOVA model with treatment as fixed effect, and baseline score, BMI, and fiber intake category as covariates. To study the effect of consumption of the study products, as a post-hoc analysis the number of responders in the three groups of the PP population was compared. Two different types of responders were defined, initial responders (subjects with an increase in stool frequency in week 1) and consistent responders (subjects with an increase in stool frequency at week 1 still present at week 3). The defined increase was at least 1 bowel movement per week. Counts and percentages of the different study groups were calculated. The percentages were compared with a Chi-square test. A *p*-value < 0.05 was considered to be significant.

For the microbiota data, bioinformatic analyses were done with the NIZO VSEARCH-based Snakemake pipeline (V3-V4 16S rRNA region). Raw sequencing data was processed into amplicon sequence variants (ASVs). The taxonomy assigned to ASVs was based on a naive Bayesian classifier (using SILVA v138). The species-level taxonomy was assigned based on exact sequence matching (100% identity). ASVs were aligned using MAFFT, and the phylogenetic tree was produced using FastTree. Counts were rarefied to 11,342 counts/sample. In the statistical analyses, age, gender, BMI, and fiber intake (low, medium, or high fiber intake) were included as covariates in the linear mixed model, with group and time as fixed effects, subject as random effect, and group*time as interaction effect. Subjects who consumed pre- or probiotic supplements were excluded from the statistical analyses. A correction for multiple testing was applied, except for *Bifidobacterium* and *Lactobacillus*, as for these two taxa a specific effect was expected as specified in the microbiota analysis plan. 

Redundancy analysis (RDA) was performed using CANOCO 5.12 (Microcomputer Power, Ithaca, NY, USA) according to the manufacturer’s instructions (ter Braak, C.J.F. and Smilauer, P. (2012) Canoco reference manual and user’s guide: software for ordination, version 5.0, Microcomputer Power) with response data (composition) transformed as Y’ = log(1000Y + 1).

## 3. Results

### 3.1. Baseline Characteristics

Between August 2020 and December 2020, 132 participants were randomly assigned to receive one of three study products: placebo (*n* = 43), 11 g GOS (*n* = 44), and 5.5 g GOS (*n* = 45).

**Figure 1:**
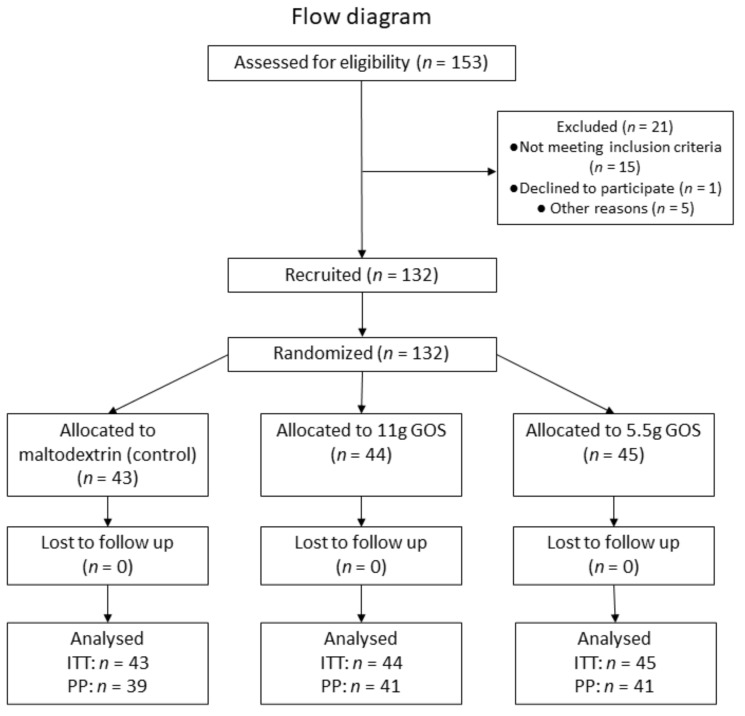
Flow diagram.

All 132 participants included in the study finished the study and were included in the intention-to-treat population (ITT). The subject flow diagram is depicted in Figure 1. The baseline characteristics of this group are shown in Table 2. At screening, all these subjects reported having less than three bowel movements per week. On average for this ITT population, straining was reported to occur in 82% of the defecations, lumpy or hard stool in 67% of the defecations, sensation of incomplete evacuation in 73% of the defecations, sensation of obstructions in 61% of the defecations, and manual maneuvers in 28% of the defecations.

From the ITT population, 121 participants were compliant to the protocol and were included in the per protocol population (PP). Reasons for exclusion of PP population were extensive weight gain, stomach flu, food poisoning, antibiotic use, missing baseline values, change in eating pattern, and a compliance of <90%. The study products were well tolerated, and no serious adverse events were reported that could have been associated with the intake of the study products.

### 3.2. Stool Characteristics

**Figure 2:**
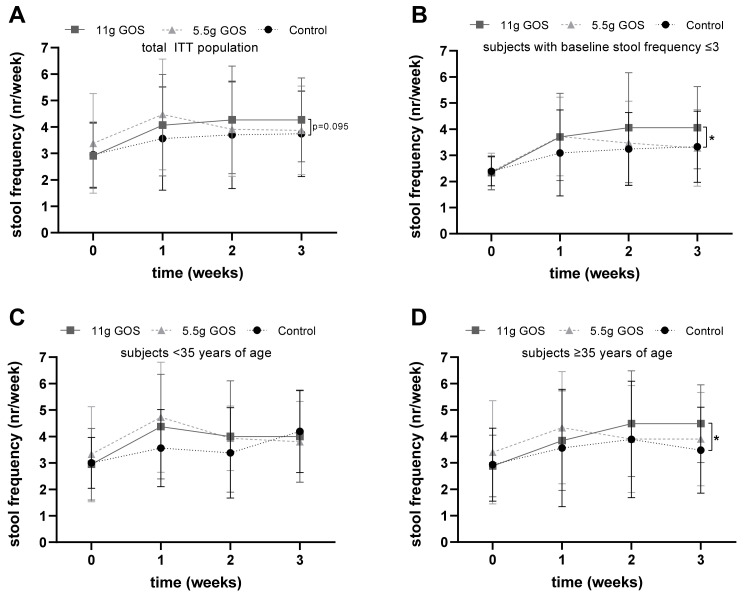
Stool frequency during the intervention period of ITT population, (**A**) Overview of the total ITT population for the ● control group (*n* = 43), ν 11 g GOS group (*n* = 44), and the 5.5 g GOS group (*n* = 45). Mixed model analysis showed a trend towards significance (*p* = 0.095) between the control and 11 g GOS groups at week 3. (**B**) Subjects from ITT population with a basal stool frequency ≤3, for the ● control group (*n* = 33), ν 11 g GOS group (*n* = 33), and the 5.5 g GOS group (*n* = 32). Mixed model analysis showed a significant difference * (*p* = 0.027) between the control and 11 g GOS groups at week 3. (**C**) Subjects from ITT population <35 years of age ● control group (*n* = 16), ν 11 g GOS group (*n* = 19), and the 5.5 g GOS group (*n* = 15). No significant differences were found in the subjects <35 years of age after mixed model analysis. (**D**) Subjects ≥35 years of age for the ● control group (*n* = 27), ν 11 g GOS group (*n* = 25), and the 5.5 g GOS group (*n* = 30). Mixed model analysis showed a significant difference * (*p* = 0.010) between the control and 11 g GOS groups in subjects ≥35 years of age at week 3.

Figure 2A shows the mean response of stool frequency of the total ITT population. To answer the primary question of this study, week 3 data of the control and 11 g GOS groups were compared. The mixed model analysis showed a trend towards a significant difference between the 11 g GOS and the control group at week 3 (*p* = 0.095). No significant differences were observed in the PP population at week 3 (*p* = 0.184). To answer secondary research questions, all time points were compared to all three intervention groups in a mixed model analysis. There were no differences between the 11 g GOS group and the control group nor in the 5.5 g GOS group and the control group.

Subjects were selected based on self-reported constipation, including the Rome IV criteria of less than three bowel movements per week. It was found that at baseline 59% of the subjects had a higher stool frequency (ranging from 3–10 bowel movements per week) than reported at screening. To study the effect of the study products in the actual constipated subjects (defined as a less than three bowel movements per week), a subgroup analysis was performed. The mixed model analysis for this subgroup showed a trend towards significantly increased stool frequency in the 11 g GOS group compared to the control group (*p* = 0.071). As the number of subjects with a stool frequency below three was very low (*n* = 54), a post-hoc analysis was performed on the subgroup of subjects with a low stool frequency at baseline (*n* = 98) (defined as ≤3 bowel movements per week). The other Rome IV criteria that were used for screening for this subgroup were on average: straining in 81% of the defecations, lumpy or hard stool in 68% of the defecations, sensation of incompletion in 74% of the defecations, sensation of obstruction in 62% of the defecations, and manual maneuvers in 33% of the defecations. Therefore, this subgroup was considered as suffering from constipation-related issues. Figure 2B shows the results of this subgroup with ≤3 bowel movements per week. The mixed model analysis showed a significantly increased stool frequency in the 11 g GOS group compared to the control group (*p* = 0.027).

To be able to study the effect in young adults versus middle-aged adults in this study population, the total study population was divided into two groups: <35 years of age at baseline, or 35 years and older at baseline. Baseline characteristics of these subgroups are shown in Supplemental Table S1. Figure 2C,D show the stool frequency of those subgroups in time. The mixed model analysis showed a significantly higher stool frequency between the 11 g GOS groups and the control group in the subjects aged 35 years and older (*p* = 0.010). This was not found for the subjects below 35 years of age (control vs. 11 g GOS, *p* = 0.603).

Table 3 shows the outcomes of the stool consistency (weekly average) during the study period. A score of two reflects *lumpy, hard-to-pass sausage-shaped* and a score of three reflects *stool with cracks on its surface*. No significant differences were observed between the intervention groups during the study.

**Figure 3:**
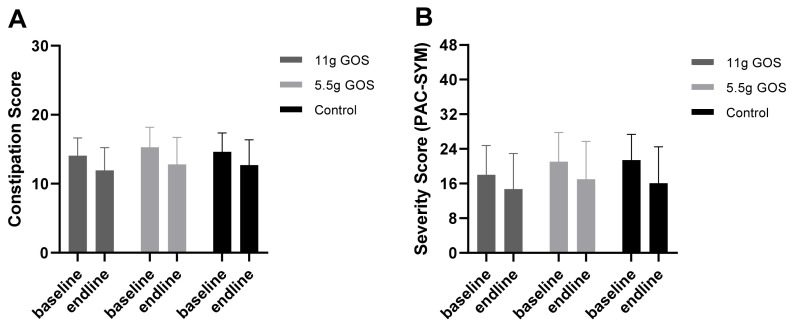
(**A**) Constipation score (based on Agachan et al., 1997) (ranging from 0–30) and (**B**) symptom severity score (PAC-SYM) (ranging from 0–48) of ITT population at baseline and after the intervention period. The ANCOVA did not show any differences in Constipation Score between 11 g GOS and control (*p* = 0.529), nor between 5.5 g GOS and control (*p* = 0.717). The ANCOVA did not show any differences in PAC-SYM scores between 11 g GOS and control (*p* = 0.801), nor between 5.5 g GOS and control (*p* = 0.446).

Figure 3 shows the outcomes of the Constipation Scoring system and the PAC-SYM questionnaire. The scores seem to decrease (i.e., improve) during the intervention in the three groups. However, no differences were found between the GOS groups and the control.

Table 4 presents the percentage and number of initial and consistent responders in the different intervention groups of the PP population. Initial responders were defined as subjects with an increase in stool frequency in week 1. Subjects with an increase in stool frequency at week 1 which remained until week 3 were seen as consistent responders. Baseline characteristics of both responder groups are shown in Supplemental Table S2. The percentage of both types of responders was found to be higher in the 11 g GOS group compared to the control group (respectively, *p* = 0.012 and *p* = 0.039). The 5.5 g GOS group was not different from the control group.

### 3.3. Fecal Microbiota

In total, 263 samples were collected and sequenced. Of those samples, three samples were excluded due to low quality. Stool samples of four subjects were excluded from analysis due to consumption of pre- and probiotics other than the supplied products. 

**Figure 4:**
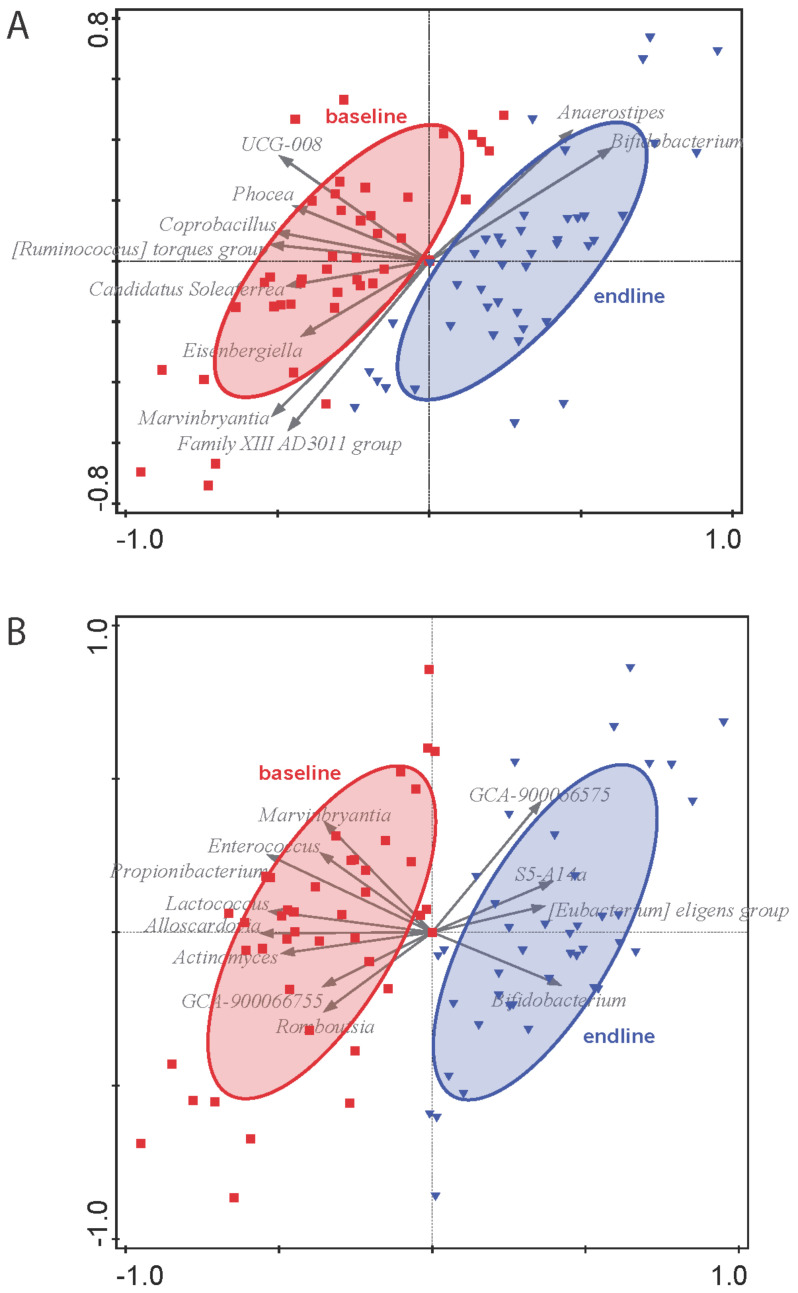
Partial RDA of (**A**) 11 g GOS group in time and (**B**) 5.5 g GOS group in time. Red squares represent baseline samples, blue triangles represent endline samples. Envelopes are 2D-normal-based ellipses at the 66% level. Grey arrows represent the genera with the best fit in the ordination space on the horizontal axis.

Figure 4 illustrates the outcome of the partial RDA on the impact of the interventions on the microbiota composition on the genus level corrected for subject. A clear distinction in time can be observed for both the 11 g GOS (Figure 4A) and 5.5 g GOS (Figure 4B) groups. For the 11 g GOS group, the explained variation is 4.29% for the total composition (*p* = 0.008). The genera that contribute most prominently to this change in time are *Bifidobacterium* and *Anaerostipes*. For the 5.5 g GOS group, the explained variation is somewhat lower at 3.51% (*p* = 0.038), but also *Bifidobacterium* being the main genus that contributes to the change in microbiota composition during the intervention. The control group did not show a significant overall effect in time on microbiota composition (*p* = 0.26, data not shown).

**Figure 5:**
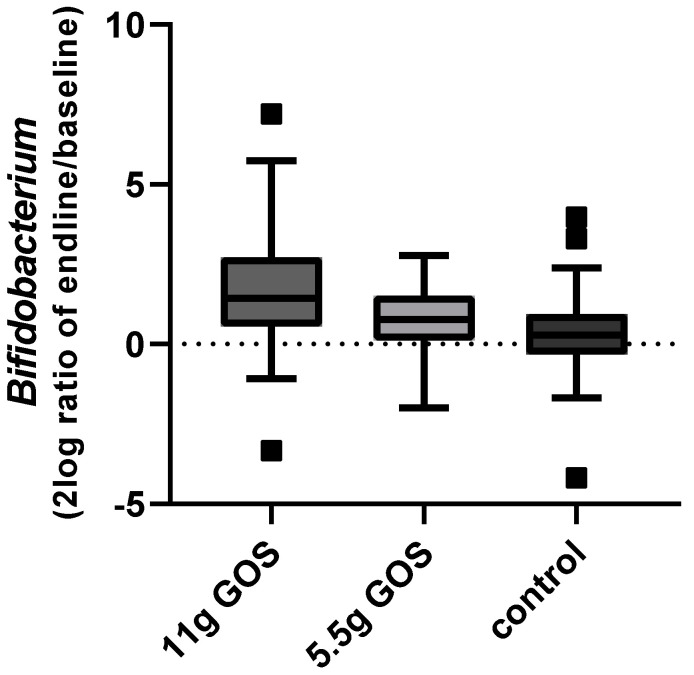
Change in *Bifidobacterium* over time (2log ratio of relative abundance at endline (week 3) and at baseline) for the different study groups. Boxplots are displayed as Tukey whiskers.

Figure 5 shows the change of *Bifidobacterium* during the intervention, displayed as 2log ratio of relative abundance at endline and baseline. The linear mixed model indicated that compared to baseline the relative abundance of *Bifidobacterium* significantly increased (*p* < 0.001), from 10.9% to 23.9% for the 11 g GOS group. For the 5.5 g GOS group, a non-significant change (*p* = 0.16) from 11.6% to 19.1% was found. Both Figure 4 and Figure 5 show a dose-response effect of GOS on *Bifidobacterium* levels, with a significant increase for the 11 g dose. 

**Figure 6:**
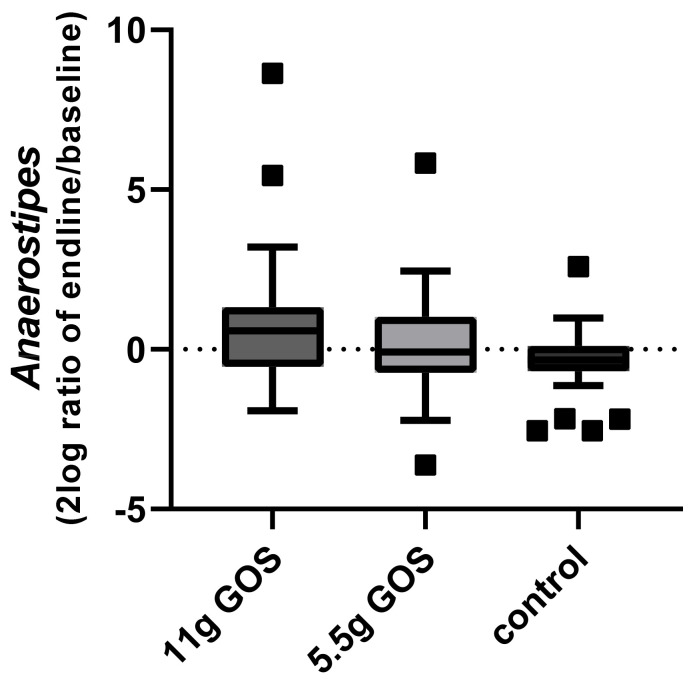
Change in *Anaerostipes* over time (2log ratio of relative abundance at endline (week 3) and at baseline) for the different study groups. Boxplots are displayed as Tukey whiskers.

Another genus affected by the intervention of 11 g GOS is *Anaerostipes*, according to the outcome of the linear mixed model (11 g GOS: adjusted *p* = 0.03, 5.5 g GOS: adjusted *p* = 0.6). The change over time of *Anaerostipes* is displayed in Figure 6. This effect was mainly explained by the increase in the species *Anaerostipes hadrus*, as this was also shown to be significantly higher in week 3 upon the consumption of 11 g GOS as indicated by the linear mixed model (11 g GOS: adjusted *p* = 0.03, 5.5 g GOS: adjusted *p* = 0.57). 

Overall microbiota composition was linked to age, average stool consistency score, and stool frequency as tested with RDA. Age explained 1.84% (adjusted *p* = 0.02) of the variation, average stool consistency score 1.50% (adjusted *p* = 0.03), and stool frequency 1.21% (adjusted *p* = 0.05).

Fecal SCFA concentrations did not significantly differ after three weeks of intervention between the groups (data not shown).

## 4. Discussion

To the best of our knowledge, this is the first study to investigate the effect of prebiotic GOS in an adult population from 18–56 years with self-reported constipation. This randomized placebo-controlled study documents a clinically relevant effect of GOS in those with low stool frequency. This study has shown that the daily consumption of 11 g of GOS tends to increase stool frequency in adults with self-reported constipation. Stool frequency was significantly increased in the subgroup of adults with a stool frequency of less than or equal to three bowel movements per week (with 1.3 bowel movement), and in a subgroup of middle-aged (35–56 years) adults with self-reported constipation (with 1.1 bowel movement). 

This work extends the existing knowledge of GOS by using validated methods. Previous studies focused on older adults with constipation, with ages ranging from 60 to 92 years. In the present study, adults from 18–56 years were included. This research has also provided additional evidence with respect to age. A subgroup analysis in which the subjects were split based on younger adults and middle-aged adults clearly showed that the effect on stool frequency was mainly present in the subjects aged 35 years or above. 

The current findings of GOS supplementation on stool frequency are of clinical interest, showing at least one additional defecation per week crossing the threshold of three bowel movements per week. These data indicate a normalization of bowel function [31].

In addition, the present study contributes to the identification of responders. Both initial and consistent responders were characterized by lower stool frequency scores measured at baseline. The response rates in the control and GOS groups implicate that despite a high (placebo) response rate in the control group, there is still a higher chance to become a responder when consuming GOS. 

The present interventions did not show differences in stool consistency scores. Previous smaller studies in older adults of 60 years and beyond showed that 9 to 12 g of GOS consumption per day eased defecation by improving stool frequency [18,19] or a reduction in defecation difficulty [21]. In terms of stool consistency, no change [18] or a tendency towards softer stools [19] was reported. Overall, the assessment methods used in these studies differed from the present study. In contrast to previous studies, in the current study validated questionnaires were applied including the Bristol Stool Scale (BSS). These BSS baseline scores were between 2 and 3 on a scale of 7. As a BSS score of 4 is seen as normal, this indicates that the stool consistency was not very hard to pass at the start of the intervention, and therefore there was not much room for improvement during the intervention. 

The majority of participants in this study were female. This might be explained by the fact that there are gender differences in self-reported constipation. Women may seem to experience a number of constipation symptoms and abnormal bowel habits more frequently than men [32]. In the current study, an effect on stool frequency in middle-aged adults was observed, but not in the younger adults. As studied in healthy volunteers, in women aging seems to impact intestinal transit significantly [32]. In men, the colonic mean transit time was unaffected by age, but middle-aged women had a significantly slower colonic transit time than young women [32]. There could also be underlying age-related changes in the intestinal microbial composition involved [33]. In the present study we noticed an age effect on overall microbial composition at baseline. All of this remains speculative, as the current research was not designed to evaluate mechanisms related to the differences in stool frequency, but in future studies it would be of interest to measure transit time and lifestyle habits including exercise to be able to investigate these factors in constipation.

In the current study, a clear dose-response effect of GOS was shown on the *Bifidobacterium* genus. This could be the result of direct fermentation and/or cross-feeding interactions [34]. Bifidobacteria are Gram-positive, anaerobic, saccharolytic bacteria that belong to the phylum Actinobacteria [34]. The presence of the *Bifidobacterium* genus in the gut has been correlated to gut homeostasis and a decreased number of *Bifidobacterium* species has been associated with antibiotic-associated diarrhea, IBS, and IBD [35]. Overall, it has been well established that bifidobacteria confer health-promoting benefits to humans [36]. An important function of *Bifidobacterium* linked to these benefits is degrading and fermenting carbohydrates which results in the production of SCFA [34,37].

Another species that was found to be significantly increased by GOS was *Anaerostipes hadrus*, which has been identified as a dominant butryate-producing species in a healthy human colon [38,39]. *Anaerostipes hadrus* has been associated with maintaining intestinal metabolic balance due to its ability to utilize glucose and fermentation intermediates acetate and lactate to form butyrate and hydrogen. Indeed, significantly higher abundance has been reported in fecal samples from healthy adults compared to those suffering from colorectal cancer [40] or in people suffering from IBS [41]. In line with findings in the current study, *Anareostipes* was shown to be induced by fructan types of oligosaccharides in adults with mild constipation [42] and healthy subjects [43], respectively, consuming 12 or 20 g of inulin per day.

Several studies have investigated differences in gut microbiota composition in constipated adults versus healthy controls with conflicting results, possibly due to differences in methods of assessment [44]. Two studies indicated that the abundance of *Bifidobacterium* decreased in constipated adults [23,24], but this could also have been related to aging, which has been linked with lower *Bifidobacterium* levels. Parthasarthy et al. (2016) concluded that constipated patients had a unique profile of colonic microbiota that discriminated health with constipation with an accuracy of 94% independent of transit time or diet [25]. In the current study, an association between overall gut microbiome at baseline and clinical measurements as stool frequency and consistency were identified. These findings shed more light on the relationship between gut microbiota and constipation.

Despite differences in fecal microbiota composition related to *Bifidobacterium* and *Anaerostipus* abundance between subjects consuming either GOS or control supplements, in the current study there were no significant differences shown in fecal SCFA. This is in line with findings of Wilms et al., who did not find a change in fecal SCFA concentrations after four weeks of GOS supplementation, despite showing differences in bifidobacterial abundance [45]. This could be related to the short intervention period applied. Moreover, quantification of human intestinal SCFA in fecal samples has its limitations as most SCFA produced in the colon are absorbed or metabolized by other gut bacteria. As such, data interpretation of fecal SCFA should be completed with some caution [46]. Further research exploring longer term intervention with GOS and using the analysis of volatile organic compounds (VOCs) in exhaled breath may extend the knowledge about the role of SCFA in the dietary management of constipated adults with galacto-oligosaccharides [47,48]. Finally, it would be interesting to assess the role of mucosal microbiota by using colonic tissue samples. This may provide new leads to unravel the relationship between gut microbiota and the dietary management of constipation.

### Strengths and Limitations of the Present Study

A key strength of this study was the inclusion of a larger sample size compared to previous studies. This allowed for the analysis of different sub-groups by which we could dig deeper into which part of the general adult population with self-reported constipation the intervention was most effective. Another strength of this study is the application of multiple validated questionnaires and methods to measure the impact on stool characteristics. The most important limitation of this study lies in the fact that although subjects reported being constipated at screening, 59% of the included subjects actually had a higher score than three bowel movements on a weekly basis at the start of the intervention. Knowing that a score of three is defined as the low-end range of normal bowel function, a substantial number of participants, thus, did not formally suffer from actual constipation. In the actual constipated subjects (with a stool frequency lower than three), a trend towards significance was found. A significant effect might have been found with a larger group of constipated subjects.

In this study, we found a substantial response rate in the control group with 35.9% consistent responders, despite traditional methods of reducing placebo response in clinical trials [49]. This placebo response rate is in line with data from a meta-analysis of RCTs for GI conditions showing a placebo response rate of 36.5% [50]. A run-in phase with the control product preceding randomization combined with a baseline measurement as inclusion criteria could have been helpful in lowering placebo response and identifying the true constipated subjects.

## 5. Conclusions

This study demonstrates a trend towards an increased stool frequency after three weeks of intervention with 11 g of GOS in adults with self-reported constipation, and a significant increase in adults with low stool frequency (≤3 bowel movements per week). Furthermore, in a subgroup of middle-aged adults (35 years and older), consumption of 11 g of GOS significantly increased the number of bowel movements per week. A clear dose-response effect of GOS was found on fecal *Bifidobacterium*. Overall, these findings contribute to the understanding of the dietary management of constipation with prebiotics.

## Figures and Tables

**Table 1:** Nutrient composition of the study products (per sachet, daily amount).

	Control	11 g GOS *	5.5 g GOS *
Amount (g)	15.1	15.1	7.5
Energy (kcal)	58.3	32.2	22.0
Fat (g)	0	0	0
Carbohydrates (g)	14.5	14.6	7.3
Maltodextrin (g)	13.6	-	-
Galacto-oligosaccharides, (g) (≥DP2) **	0	10.4	5.2
Lactose (g)	0.9	3.5	1.7
Glucose (g)		0.6	0.3
Galactose (g)		0.06	0.03
Protein (g)	<0.1	0	0
Dietary fiber (g) (≥DP3) *	0	7.2	3.6
Salt (g)	<0.005	0	0

* Biotis^TM^ GOS was used ** DP: degree of polymerization. According to EU legislation (EU/1169/2011) from ≥DP3 can be called a fiber. Therefore, not all GOS are included in the fiber count.

**Table 2:** Baseline characteristics of ITT Population (presented as means ± SE or percentage). BSS = Bristol Stool Chart.

	Control	11 g GOS	5.5 g GOS
*n*	43	44	45
Gender (% female)	93.0%	90.9%	97.8%
Age (years)	38.8 ± 13.3	37.3 ± 11.5	38.9 ± 11.8
BMI (kg/m^2^)	24.4 ± 2.2	24.2 ± 2.8	24.8 ± 2.8
Weight (kg)	70.8 ± 6.9	70.5 ± 9.8	72.0 ± 10.8
Waist circumference	86.5 ± 7.9	84.1 ± 9.6	86.8 ± 8.9
Hip circumference	103.3 ± 4.7	102.0 ± 6.8	103.4 ± 7.2
Fiber intake category (% low)	58.1%	59.1%	60.0%
Stool frequency (bowel movements/week)	3.0 ± 1.2	2.9 ± 1.2	3.4 ± 1.9
Stool consistency (BSS score)	2.7 ± 1.3	2.7 ± 1.2	2.8 ± 1.3

**Table 3:** Average stool consistency per week (Bristol Stool Scale Score) of ITT Population during the intervention (mean ± SD).

	11 g GOS	5.5 g GOS	Control	*p*-Value 11 g GOS vs. Control	*p*-Value 5.5 g GOS vs. Control
*n*	43	45	42		
Baseline	2.7 ± 1.2	2.8 ± 1.3	2.7 ± 1.3	0.791	0.666
Week 1	3.3 ± 1.4	2.8 ± 1.1	3.1 ± 1.4	0.395	0.315
Week 2	3.0 ± 1.2	2.9 ± 1.2	2.7 ± 1.1	0.133	0.411
Week 3	3.1 ± 1.1	2.8 ± 1.3	2.9 ± 1.3	0.371	0.621

**Table 4:** Initial responders (subjects with an increase in stool frequency in week 1) and consistent responders (subjects with an increase in stool frequency at week 1 still present at week 3) from the PP population in all three intervention groups (percentages and counts). The *p*-value represents the outcome of the Chi-square test in which the three intervention groups were compared.

	11 g GOS	5.5 g GOS	Control	*p*-Value
Initial responders	80.5% (*n* = 33) ^a^	65.9% (*n* = 27) ^a,b^	48.7% (*n* = 19) ^b^	0.012
Consistent responders	63.4% (*n* = 26) ^a^	43.9% (*n* = 18) ^a,b^	35.9% (*n* = 14) ^b^	0.039

^a,b^ Different letters in superscript indicate differences between intervention groups.

## Data Availability

The data in this study are not publicly available but can be requested from the corresponding author.

## Supplementary Materials

The following are available online at https://www.mdpi.com/article/10.3390/nu14020309/s1, Figure S1: Schedule of Assessment, Table S1: Baseline characteristics of subgroups based on age in ITT Population, Table S2: Baseline characteristics of responders and non-responders in the ITT Population.
