# Supplementary material for: Prebiotic Galacto-Oligosaccharides Impact Stool Frequency and Fecal Microbiota in Self-Reported Constipated Adults: A Randomized Clinical Trial
Source: Nutrients. 2022 Jan 12;14(2):309. doi: 10.3390/nu14020309 (PMC8780623; doi:10.3390/nu14020309)
Supplement: Supplementary file 1 [file nutrients-14-00309-s001.zip › nutrients-1523158-supplementary-done.pdf]

nutrients

MDPI

Supplementary Materials

Activities	Screening	Visit 1	Baseline week							Run-in period				Intervention period																					Visit 2
														Week 1							Week 2							Week 3							
Day	< -7	-7	-7	-6	-5	-4	-3	-2	-1	1	2	3	4	5	6	7	8	9	10	11	12	13	14	15	16	17	18	19	20	21	22	23	24	25	26
**Screening**																																			
Informed consent	x																																		
Screening questionnaire	x																																		
Medical history	x																																		
Fiber intake FFQ	x																																		
**Visit at study site**																																			
Provision study products		x																																	
Provision fecal sample collection kits		x																																	
Weight, height, waist and hip circumference		x																																	x
Intake remaining study products																																			x
Intake fecal samples																																			x
**Study specific outcomes**																																			
Weekly questionnaire<br>- Stool frequency<br>- Stool consistency (Bristol Stool Chart)<br>- Bloating<br>- Changes in daily habits: fluid intake, smoking, alcohol use, physical activity, medication intake									x											x							x							x	
PAC-SYM questionnaire									x																									x	
Constipation Scoring System questionnaire									x																									x	
Fecal sample collection									x																									x	
**Monitoring**																																			
Study product intake																				x							x							x	
Adverse Events/Serious Adverse Events	x	x	x	x	x	x	x	x	x	x	x	x	x	x	x	x	x	x	x	x	x	x	x	x	x	x	x	x	x	x	x	x	x	x	

Figure S1. Schedule of Assessment.

Table S1. Baseline characteristics of subgroups based on age in ITT Population (in mean ± SD or median , IQR).

	**<35 years of age**	**≥35 years of age**
n	50	82
Gender (% female)	92.0%	95.1%
Age (years)	24.7 ± 4.5	46.6 ± 6.4
BMI (kg/m2)	23.3 ± 2.2	24.0 ± 2.3
Weight (kg)	70.2 , 11.0	71.1 ± 8.7
Waist circumference	82.2 ± 8.6	88.0 ± 8.3
Hip circumference	102.8 ± 6.6	103.0 ± 6.2
Fiber intake category (% low)	56.0%	61.0%
Stool frequency	3.0 , 1	3.0 , 2
Stool consistency	3.1 ± 1.3	2.3 , 1.3

Table S2. Baseline characteristics of responders and non-responders in the ITT Population (in mean ± SD or median , IQR). Initial responders: subjects with an increase in stool frequency in week 1. Consistent responders: subjects with an increase in stool frequency at week 1 still present at week 3.

	**Initial responders**		**Consistent responders**	
	yes	no	yes	no
n	49	83	60	72
Gender (% female)	91.6%	98.0%	90.0%	97.2%
Age (years)	39.0 , 25	43.0 , 17	37.5 , 24	43.0 , 21
BMI (kg/m2)	23.8 ± 2.3	23.5 ± 2.4	23.8 ± 2.3	23.6 ± 2.3
Weight (kg)	72.0 ± 9.8	69.6 ± 8.3	72.2 ± 9.7	70.2 ± 8.9
Waist circumference	86.0 ± 9.1	85.5 ± 8.4	86.4 ± 9.1	85.3 ± 8.7
Hip circumference	103.0 ± 6.6	102.8 ± 5.9	103.4 ± 6.6	102.5 ± 6.1
Fiber intake category (% low)	59.0%	59.2%	56.7%	61.1%
Stool frequency	2.8 ± 1.3	3.5 ± 1.7	2.0 , 1	3.0 , 2
Stool consistency	2.7 , 1.8	2.5 , 1.4	2.5 , 1.5	2.5 , 1.3
